# Multimorbidity among adult Indians –prevaLence, incidence, risk fActors and economic burdeN: the MILAN cohort study protocol

**DOI:** 10.1136/bmjopen-2025-100853

**Published:** 2025-10-29

**Authors:** Priti Gupta, Sailesh Mohan, Sanghamitra Pati, Colin McCowan, Dimple Kondal, Viswanathan Mohan, Dorairaj Prabhakaran

**Affiliations:** 1 Centre for Chronic Disease Control, New Delhi, Delhi, India; 2 Department of Health Research, Indian Council of Medical Research Chandrasekharpur, Bhubaneswar, Orissa, India; 3 University of St. Andrews, St Andrews, UK; 4 Madras Diabetes Research Foundation (ICMR-Collaborating Centre of Excellence), Chennai, Tamil Nadu, India

**Keywords:** Multimorbidity, EPIDEMIOLOGIC STUDIES, Chronic Disease

## Abstract

**Abstract:**

**Introduction:**

Multimorbidity or multiple long-term conditions (MLTCs) are defined as the coexistence of two or more chronic conditions in an individual. With increased longevity and the rising burden of chronic non-communicable diseases (NCDs), multimorbidity is becoming the norm. Although more prevalent in older populations and people with low socio-economic status, multimorbidity is rapidly rising in the younger age groups. Accurate data on its incidence and health and economic impacts, ie, disability-adjusted life years (DALY) lost and quality-adjusted life year (QALY) are not available for the Indian population. The objective of this study is to determine the incidence and predictors of multimorbidity, the longitudinal trends, the common clusters of conditions and the health and economic impact of multimorbidity among adult Indians aged ≥40 years.

**Methods and analysis:**

12 229 participants (≥40 years) from the population-based cohort, titled the Centre for cArdiometabolic Risk Reduction in South-Asia (CARRS) cohort, from Delhi and Chennai will be recruited. CARRS is an existing adult urban cohort which is well characterised, deeply phenotyped and geocoded with bio-banked samples. They will be followed up longitudinally twice during 2023–2025. Information will be collected on common NCD risk factors (physical inactivity, tobacco and alcohol use), disability, frailty and treatment costs. We will also perform anthropometric and blood pressure measurements on all participants as well as biomarker assessments on a sub-sample of 2300.

**Ethics and dissemination:**

Ethics approval has been obtained from the ethics committees of the Centre for Chronic Disease Control (CCDC) (Institutional Review Board (IRB) 00006330) and the Madras Diabetes Research Foundation (IRB no. IRB00002640). Key findings from the study will be published in national and international peer-reviewed journals. Results will also be presented at various academic conferences to engage with the broader research community. A final report will be submitted to the funding agency upon completion of the fellowship. De-identified data will be securely stored at the CCDC. Access to the data will be available upon request to the principal investigator.

**STRENGTHS AND LIMITATIONS OF THIS STUDY**

- This study has a cohort study design which will enable us to generate data on the incidence of multiple long-term conditions in India.
- Multimorbidity will be assessed holistically using standard definitions.
- The study population is from Delhi and Chennai, two major metropolitans’ cities of India, so the study is not nationally representative.
- The sample size for the secondary objective, that is, cluster analysis will be sub-optimal.

## Background

 Multimorbidity is defined as the coexistence of ≥two chronic conditions.^1^ With increased longevity and the concomitant rising burden of chronic non-communicable diseases,^2 3^ multimorbidity is emerging as a high priority public health concern globally. However, due to the use of inconsistent definitions and classifications in currently available studies, determining the actual burden of multimorbidity is challenging.^4 5^ Most studies investigating the burden are from high-income countries (HICs) and among those aged ≥60 years and focus on the prevalence of burden. However, there is a strong emerging evidence base, which indicates that multimorbidity is a lifelong problem that is increasingly affecting large numbers of younger people as well, particularly in low- to middle-income countries (LMICs).^6 7^ Among those with lower socio-economic status, multimorbidity occurs more frequently at earlier ages as compared with those in the higher-income groups.^8^ Therefore, multimorbidity presents a challenge for both HICs and LMICs. Currently, data on the prevalence and determinants of multimorbidity are insufficient in many parts of the world, while incidence data are deficient globally.

The most prevalent patterns of the disease within multimorbidity, according to a recent systematic review from Asia, include cardiovascular and metabolic illness, mental health issues, degenerative diseases, lung diseases and cancers.^9^ According to a recent study using nationally representative data, the most frequent combinations of multiple morbidities among young Indians (under 50 years of age) are obesity and anaemia, hypertension and obesity, and hypertension and anaemia.^10^ The most frequent patterns, according to another systematic study from India, include hypertensive diseases associated with diabetes mellitus, chronic joint pain, heart disease and metabolic disorder.^11^ Another study using Longitudinal Ageing Study in India (LASI) 2017–2018 data has highlighted hypertension in many of the common patterns of multimorbidity in India.^12^ Age, female gender, lower socio-economic status and physical inactivity were reported as the main risk factors for multimorbidity.^13 14^

This study will fill the critical existing evidence gap regarding the incidence of multimorbidity, common multimorbidity clusters, associated risk factors, health (disability-adjusted life years (DALY)) and economic impacts (healthcare costs) from two different regions of India using the Health Data Research, UK (HDR, UK) multimorbidity framework.^15^ This study will use globally accepted definitions, validated tools and measurement strategies as well as include those ≥40 years who have been previously understudied in most available studies.

The overall aim of this study is to describe multimorbidity and its determinants in a large representative Indian population aged ≥40 years. It will determine the incidence of multimorbidity, the longitudinal trends, the common clusters of conditions and the health and economic impact of multimorbidity.

## Methods

### Study population and sampling strategy

The parent study Centre for cArdiometabolic Risk Reduction in South-Asia (CARRS): this is a community-based cohort study, composed of two separate, independently sampled cohorts, of adults aged 20–99 years recruited from two large Indian cities (Delhi and Chennai), using identical measures, and surveyed at baseline (CARRS cohort-1 (2010–2011) and CARRS cohort-2 (2014–2015)), with regular periodic longitudinal follow-ups. In this study, we will be following both the cohorts, recruited in 2010–2011 and 2014–2015 from Delhi and Chennai. Multi-stage cluster random sampling was used, followed by the use of the Kish method for within-household participant selection.^16^ The primary aim of the CARRS cohort was to measure the prevalence and risk factors of cardiometabolic diseases and study cardiovascular disease trends.^17 18^

### Study measures

We will use the existing CARRS study’s measures for the assessment of socio-demographic variables, risk factors (alcohol, tobacco, physical inactivity), depression (via PHQ-9), quality of life, healthcare costs and causes of death (via verbal autopsy form). For multimorbidity, disability and frailty assessments, we will use previously validated questionnaires. Details of these study measures are provided in table 1. All the study questionnaires will be translated into the local language, that is, Hindi for Delhi and Tamil for Chennai.

**Table 1:** Summary of the study measures in the parent and proposed study

	Parent study (CARRS cohort)	Proposed study
Study participants	Adults aged ≥20 years residing in the sampled urban areas	All adults aged ≥40 years who continue to be followed up in the cohort
Socio-demographic variables	Age, gender, educational status, occupation, marital status, wealth index (based on income and household assets)	
Risk factors	Tobacco and alcohol use, unhealthy diet, physical inactivity (based on the comparative risk assessment framework of the Global Burden of Disease Project)	
Anthropometric parameters	Height (using SECA portable stadiometer), weight and body fat distribution (bio-impedance using Tanita BC-418), waist and hip circumference (using a non-stretch measuring tape), blood pressure measurements (using electronic sphygmomanometer OmronHEM-7080)	
Biochemical parameters	Blood: fasting blood sugar, postprandial blood sugar, HbA1c, total cholesterol, high-density lipoprotein, low-density lipoprotein, very-low-density lipoprotein and creatinine	In addition to data collected in the parent study, data on thyroid-stimulating hormone, T3, T4 and hepatitis B will also be collected
Healthcare costs	Outpatient (past 6 months) and hospitalisation (past 12 months) expense data of participants with cardiometabolic diseases	Outpatient (past 6 months) and hospitalisation (past 12 months) expense data of all participants
Depression	Using Patient Health Questionnaire-9	
Quality of life	Using EuroQol-5D (EQ-5D)	
Multimorbidity	Diabetes, hypertension, heart disease, CKD, stroke, cancer	Using Multimorbidity Assessment Questionnaire for Primary Care^23^
Disability	Not measured	Using a 12 item version of the WHO Disability Assessment Schedule 2.0
Cause of death	This will be ascertained by verbal autopsy form	
Frailty	Not measured	Fried frailty criteria

CKD, chronic kidney disease; HbA1c, glycated haemoglobin.

PHQ-9 (Cronbach’s alpha=0.77)^19^ and EQ-5 (Cronbach’s alpha=0.97)^20^ have been validated and used extensively in Indian populations, including in our cohort since 2010. Multimorbidity assessment questionnaire, WHO Disability Assessment Schedule 2.0 and Fried frailty criteria questionnaires are provided as online supplemental material. These questionnaires are validated in Indian settings.^21 22^ The multimorbidity questionnaire is also validated in the Indian setting, and trained field staff will use simple, non-technical language and visual cues where appropriate.^23^ A total of 21 long-term conditions will be assessed in this study, namely, (1) arthritis, (2) diabetes, (3) hypertension, (4) chronic obstructive lung disease, (5) acid peptic disease (gastritis), (6) chronic back ache, (7) heart disease, (8) stroke, (9) vision problem, (10) deafness, (11) dementia, (12) alcohol disorder, (13) cancer, (14) chronic kidney disease, (15) epilepsy, (16) thyroid disease, (17) tuberculosis (TB), (18) dyslipidaemia, (19) depression, (20) obesity and (21) hepatitis B.

In addition to WHODAS 2.0 for disability assessment, we are incorporating physical performance measures including: gait speed and grip strength test. These are part of our frailty assessment protocol to enhance the objectivity and robustness of physical function data. While Fried’s frailty phenotype is our primary measure, the inclusion of these physical assessments allows for broader functional characterisation in our population.

For cost data collection, we will focus on out-of-pocket expenditures for both outpatient and inpatient services, along with medicine costs. We will also ask about insurance coverage and the nature of the insurance (government vs private).

### Study design, participant selection and recruitment

This is a cohort study design. We will select all participants aged ≥40 years in 2023–2024 who continue to be followed up in the CARRS cohort. *We will collect fresh blood samples in a sub-sample of 2300 participants who will be followed up to determine the incidence and predictors of multimorbidity*. Among the CARRS cohort, a substantial proportion (approximately 17 000 participants) are aged ≥40 years. Based on prior follow-up rounds, we maintain a follow-up rate of approximately 70%, with around 30% attrition primarily due to mortality.

Participants are not reimbursed financially for their time. However, the long-standing relationship and trust built over the past decade with our cohort participants have played a vital role in maintaining high response rates and continued engagement.

**Figure 1:**
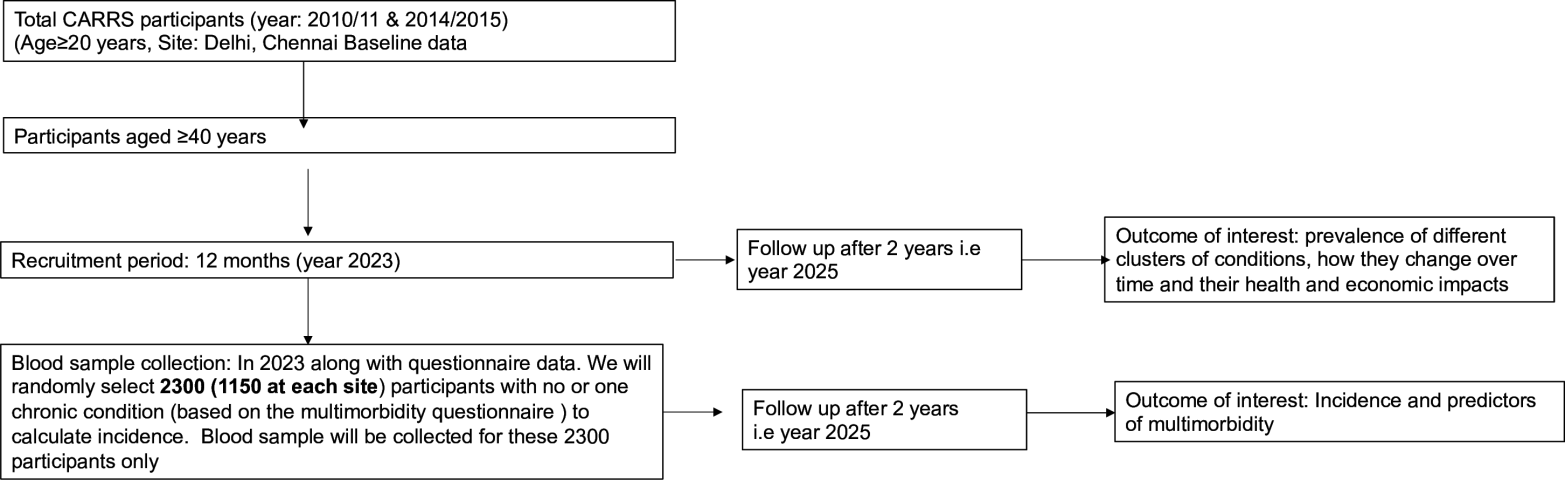
Details of participant recruitment and follow-up plans.

Figure 1 shows details of participant recruitment and follow-up plans.

### Sample size justification

*For incidence calculation*: in a community-based Malaysian study, the incidence rate for multimorbidity was 14 per 100 person-years among participants aged ≥60 years.^24^ As there is no Indian study reporting the incidence of multimorbidity, we have calculated sample size assuming different incidences, as shown in table 2. Assuming that the incidence of multimorbidity will be less in those aged ≥40 years, we will recruit around 2300 participants in Delhi and Chennai for calculating the incidence of multimorbidity, that is, 1150 participants at each site.

**Table 2:** Sample size estimation (α=0.05, power 80%)^36^

Incidence of multimorbidity per 100 person-years	Relative precision	No of cases required	Person years of observation	No of persons required*	The loss to follow-up	Sample size in each site
14	15%	170	1200	600	25%	800
12	15%	170	1400	700	25%	934
10	15%	170	1700	850	25%	1134

* follow-up period=2 years.

*For cluster analysis, health impact of multimorbidity, disability calculation:* we will include all participants aged ≥40 years who continue to be followed up in the cohort.

### Data collection, management and quality assurance

For data collection, we will leverage the already established research infrastructure of the ongoing CARRS study in the proposed study sites. All data collection will be conducted by the research team in the community using a REDCap database designed specifically for this study.^25^ The questionnaires are translated into Hindi and Tamil and will be administered by trained field researchers. None of the questionnaires are self-administered, which allows us to effectively include participants who are illiterate.

We will include built-in data checks. All data from the server will be downloaded daily to detect any irregularities in data collection in the field. In addition to examining data quality measures such as completeness of the forms, we will check the timestamp of data entry, the initials of the data collector and an average number of new records per day to monitor data processes. All the data will be monitored on a daily basis by project and data managers. All study data and laboratory samples will only be identified by a participant study ID number. All data collection will be conducted in accordance with International Conference on Harmonisation Good Clinical Practice guidelines. *Follow-up data will be collected after 2 years of the baseline data collection*. The baseline data collection will be done in 2023–2024 and the follow-up data collection in 2025–2026.

As a substantial number of participants are illiterate, we fully recognise the importance of ensuring ethical conduct and data quality, particularly for participants with limited literacy. Our process includes the following steps:

- Informed consent is obtained through a standardised procedure conducted by trained field researchers. They explain the consent form verbally in the participant’s local language, detailing the purpose, procedures, potential risks and benefits and participant rights (including withdrawal at any time).
- If preferred by the participant, a trusted family member may assist in interpreting the information.
- Consent is documented through a thumb impression and witnessed by an impartial third party.

All questionnaires are administered face-to-face by trained enumerators who read each question aloud and accurately record the participant’s responses. This method ensures that participants fully understand the questions and minimises response bias due to literacy barriers. To ensure data quality and consistency, our team implements regular training sessions, field supervision and spot-checks.

Participants were not given any incentives, as this is a long-standing cohort and participation is based on trust and engagement built over time.

### Biomarker assessments

Collection, storage and analysis of biological samples.

The Centre for Chronic Disease Control (CCDC) already has an established collaboration with Madras Diabetes Research Foundation (MDRF) who will conduct the data collection in Chennai.

Fasting blood samples (10 mL) will be collected twice during the study by trained technicians following standard procedures. We will transfer the blood samples to laboratories at CCDC in Delhi and MDRF in Chennai in iceboxes regularly for further processing. All the samples will be barcoded at the time of collection and storage.

Details of planned blood sample collection and storage are given in tables3  4.

**Table 3:** Blood sample collection and storage plan

Type of vacutainer	Volume of blood	Purpose	Storage
Plain	6 mL	Separate serum in 3 microcentrifuge tube (MCTs) for biochemical assays and future use	Vacutainer-discard after serum separation MCTs are stored at −80° for future use
EDTA	2 mL	Separate EDTA plasma in 2 MCT for HbA1c and future use	MCTs are stored at −80° for future use
Fluoride	2 mL	Separate fluoride plasma in 1 MCT for fasting blood sugar	No storage

HbA1c, glycated haemoglobin.

**Table 4:** Biological markers and their methods of analysis

Clinical parameter	Laboratory parameters	Methods used for analysis
Diabetes	FBS	Hexokinase/kinetic
	Glycated haemoglobin	High-performance liquid chromatography
Dyslipidaemia	Total cholesterol	Cholesterol oxidase peroxidase endpoint
	High-density lipoprotein cholesterol	Direct
	Low-density lipoprotein cholesterol	Friedwald formula
	Very low-density lipoprotein cholesterol	Calculation
	Triglycerides	Enzymatic methods (GPO-PAP endpoint)
Kidney diseases	Serum creatinine	Jaffe kinetic
Thyroid	TSH	Electrochemiluminescence immunoassay (ECLIA) using sandwich principle with monoclonal antibodies directed towards human TSH
	T3 & T4	T3 & T4 will be estimated by ECLIA using competitive test principle with monoclonal antibodies
Hepatitis B	Hepatitis B surface antigen assay	Enzyme immunoassay

FBS, fasting blood sugar; GPO-PAP, glycerol phosphate oxidase - peroxidase aminopyrine; TSH, thyroid-stimulating hormone.

As this study is part of a well-established cohort, we have developed infrastructure to store laboratory aliquots, including serum and plasma, for future research purposes. This biobanking effort enables longitudinal biological investigations over time.

### Patient and public involvement

In this study, patients were involved primarily at the stage of data collection through provision of informed consent. Participants were fully informed of the study objectives and procedures prior to enrolment. While patients were not involved in the development of the research question, study design or outcome measures, their contribution was integral to the conduct of the study. To ensure transparency and participant benefit, individual laboratory results were shared with participants during the study period. Dissemination of aggregate study findings will be undertaken through appropriately designed lay summaries for participants and relevant patient communities.

### Statistical analysis plan

Prevalence of multimorbidity: multimorbidity will be defined as the coexistence of two or more conditions. As the simple disease count measure fails to capture the effect of diseases on current and future functional status,^26^ we will also calculate a multimorbidity-weighted index (MWI), which weights each disease by its impact on current and future physical functioning and mortality. Weights for each disease were calculated as change in Short Form 36 physical functioning (PF) scale^27^ over a period, using data from the US nationally representative prospective studies. The range of MWI is from −9.11 to 0. Negative measures denote a decrease in physical functioning score over the time period.^26 28^ We are not directly applying the SF-36 in our cohort; instead, we are using the disease weights that were developed in the U.S. based on SF-36 data.

#### Socio-demographic and health risk factors

As potential individual-level risk factors for multimorbidity, we will examine sociodemographic factors (participant-reported age, gender, marital status, education, employment status and occupation), behavioural risk factors (tobacco and alcohol use, physical inactivity) and basic anthropometry data (height and weight). Recommended level of physical activity will be defined as walking/engaging in sport for at least 150 min in a week.

We will categorise education level (up to primary, high school or secondary, college graduation and above); and occupation (professional/medium and big business owner, skilled labourer/small business, unskilled/semiskilled, homemaker). For calculating the wealth index, we will use an index of household income, 35 amenities and assets. We will use principal components analysis methods^29^ and categorise the wealth index in tertiles.

We will estimate the prevalence and 95% CI of single morbidity and multimorbidity for the whole sample and across socio-demographic and health risk factors. For multimorbidity weight calculation, MWI using the additive model will be used.^28^ Associations of a three-level variable classifying all participants as having either no morbidities, single morbidity or multimorbidity will be estimated using the multinominal logistic regression model.

A simple matrix approach will be used to determine the patterns (dyads and triads) of different multimorbidity combinations. We identified and reported all possible combinations of two or three chronic conditions.^30^ Prevalence for a single chronic condition, combinations of two (dyad) and three chronic conditions (triad) will be calculated for the whole study sample.

### Calculation of multimorbidity incidence rate

The number of new cases of multimorbidity will be divided by the person time years of participant follow-up during the study period to calculate incidence rates with 95% CI. Person-years will be estimated from the date of enrolment that is, 2023, to the time of multimorbidity diagnosis or the last date of visit or death, whichever will be documented earlier. Age-specific incidence (cases/1000 person-years) by sex will be calculated.

New cases of multimorbidity will be defined as participants with only one or no chronic disease at baseline, that is, 2023, who have developed multimorbidity during the 2 year follow-up period. We will also report numbers separately on how many developed multimorbidity from no and single chronic disease at baseline.

*For health impacts of multimorbidity*: multimorbidity will be treated as the exposure variable with separate models for each outcome variable including quality of life, frailty, physical functioning and mortality. Analysis of covariance regression analysis will be used to estimate the mean difference in the EQ-5D visual-analogue scale and physical functioning score, adjusting for baseline values, socio-demographics and health-related risk factors of interest. We will also report health impact based on different clusters of conditions.

*The economic impact of multimorbidity*: we will use cost-of-illness methods for determining the economic burden of multimorbidity. Direct medical expenditure for each disease will be obtained by adding the expenditure on medications, lab investigations, outpatient consultations and hospitalisations. We will convert all direct costs in Indian Rupees to Purchasing Power Parity-adjusted international dollars.^31^ For all medical expenditures, each unit of resource use will be multiplied by their frequency in the last 1 year, to calculate the annual cost for that category. The expenditure on treatment will be presented as means with standard errors. Given that cost data are unlikely to be normally distributed, the bootstrap method will be used to calculate standard errors and 95% CIs for average costs.

*For DALYs and cluster analysis*: DALY calculation will estimate the years lost due to disability (YLD) and years of life lost (YLL). The YLD will be calculated by multiplying disability weights with the number of years a person lives with a disability. These weights will be derived from Global Burden of Disease 2016^32^ disability weights and will vary between 0 (no burden) and 1 (mortality). The YLL calculation will be based on the duration of disease, time of death and expected time of death.^33^ Additionally, DALYs will be computed for specific diseases and clusters of conditions to assess which disease/s cause the highest attributable disease burden. We will use latent class cluster analysis to identify the potential multimorbidity clusters.

Ethics and dissemination: ethics approval has been obtained from the ethics committees of CCDC (IRB00006330) and the MDRF (IRB no. IRB00002640). Key findings from the study will be published in national and international peer-reviewed journals. Results will also be presented at various academic conferences to engage with the broader research community. A final report will be submitted to the funding agency on completion of the fellowship. De-identified data will be securely stored at the CCDC. Access to the data will be available on request to the principal investigator.

Table 5 presents the baseline demographic details of participants, with data collected during 2023–2024. Follow-up data collection is currently ongoing.

**Table 5:** Baseline demographic details of participants

	Total	Percentage (95% CI)
	n=12 229	
Age		
30–44 years	1844	15.1 (14.5 to 15.7)
45–59 years	6305	51.6 (50.7 to 52.4)
≥60 years	4080	33.4 (32.5 to 34.2)
Gender		
Male	5626	46 (45.1 to 46.9)
Female	6603	54 (53.1 to 54.9)
Marital status		
Married	10 580	86.5 (85.9 to 87.1)
Others	2	0 (0.0 to 0.1)
Separated/divorced	84	0.7 (0.6 to 0.8)
Single	88	0.7 (0.6 to 0.9)
Widow/widower	1475	12.1 (11.5 to 12.7)
Education		
Graduation and above	2254	18.4 (17.8 to 19.1)
High school or secondary	7350	60.1 (59.2 to 61.0)
Up to primary	609	5 (4.6 to 5.4)
Illiterate	2016	16.5 (15.8 to 17.2)
Occupation		
Professional, big business, landlord, university teacher, IAS/services officer, lawyer	1198	9.8 (9.3 to 10.4)
Trained, clerical, medium business owner, middle level farmer, teacher, maintenance (in charge, personnel manager)	1736	14.2 (13.6 to 14.9)
Skilled manual labourer, small business owner, small farmer	1519	12.4 (11.9 to 13.0)
Semi-skilled manual labourer, marginal landowner, rickshaw driver, army jawan, carpenter, fitter	661	5.4 (5.0 to 5.8)
Unskilled manual labourer, landless labourer	183	1.5 (1.3 to 1.7)
Homemaker	5117	41.9 (41.0 to 42.8)
Retired/student/unemployed	1749	14.7 (14.1 to 15.3)
Physical activity		
Low	5594	45.7 (44.9 to 46.6)
Moderate	1007	8.2 (7.8 to 8.7)
Severe	937	7.7 (7.2 to 8.1)
Missing	4691	38.4 (37.5 to 39.2)
Wealth index		
Low	4410	36.1 (35.2 to 36.9)
Medium	2307	18.9 (18.2 to 19.6)
High	3321	27.2 (26.4 to 28.0)
Missing	2191	17.9 (17.2 to 18.6)
Current alcohol use		
No	9792	80.1 (79.4 to 80.8)
Yes	2437	19.9 (19.2 to 20.6)
Current tobacco use		
No	10 047	82.2 (81.5 to 82.8)
Yes	2182	17.8 (17.2 to 18.5)
BMI		
<18.5 underweight	334	2.7 (2.5 to 3.0)
18.5–22.99 normal	1821	14.9 (14.3 to 15.5)
23–24.99 overweight	1562	12.8 (12.2 to 13.4)
≥25 obese	7073	57.8 (57.0 to 58.7)
Missing	1439	11.8 (11.2 to 12.4)
Abdominal obesity		
Normal	2596	21.2 (20.5 to 22.0)
Central obesity	9633	78.8 (78.0 to 79.5)

BMI, body mass index; IAS, Indian Administrative Service.

## Discussion

This study will provide hitherto unavailable contemporary data on the prevalence, incidence, associated risk factors, longitudinal trends, the health and economic impacts of multimorbidity in India. Notably, these findings will help identify the key determinants and causal pathways of multimorbidity and support the development of contextually relevant, resource-sensitive preventive and management strategies tailored to India and likely for other countries facing similar challenges.

To the best of our knowledge, this is the first longitudinal study in India focusing on multimorbidity in the younger and economically productive population. Importantly, the study’s design will facilitate the calculation of multimorbidity incidence, the establishment of temporal relationships between risk factors and the identification of causal links between behavioural, socio-demographic and biological factors, as well as different clusters of conditions. By collecting longitudinal data, including biomarkers, this study will provide a robust and efficient means of identifying the main causal factors for multimorbidity.

Multimorbidity will be assessed using a validated questionnaire tailored to the Indian context. The most recent Delphi study recommends 24 ‘always include’ diseases and 35 ‘usually include’ diseases in defining multimorbidity, most of which are covered in this study.^34^ Conditions not included in the list, such as Parkinson’s disease and Addison’s disease, were excluded due to their complexity for assessment in a population-based survey among those with low literacy levels and limited medical documentation. However, participants have been asked if they have any conditions outside the scope of the multimorbidity questionnaire, which should address these gaps. While most population-based studies on multimorbidity rely on self-reported data, this study will incorporate objective biochemical measurements, enabling the identification of asymptomatic health issues and providing a more accurate assessment of disease burden over time.

Using the HDR UK multimorbidity framework, this study will apply a consistent definition of multimorbidity, addressing the common issue of inconsistent reporting across studies. This standardised approach will facilitate future comparative analyses by integrating rigorously collected data from various settings and countries, enabling the identification of temporal trends. It will also contribute to a larger dataset on the burden and determinants of multimorbidity, which will support possible national, regional and global analyses aimed at improving the associated public health management strategies.

By selecting sites from both North and South India, the study will offer reliable data on multimorbidity clusters across diverse population subgroups and urban settings. Further, by analysing healthcare costs associated with multimorbidity across different regions and age groups, the study will provide valuable insights for policymakers regarding health service planning, provision and resource allocation. Identifying common multimorbidity clusters will also facilitate early screening efforts, leading to timely diagnoses and appropriate management.

Notably, this study will establish a multimorbidity cohort from both Northern and Southern India, laying a robust foundation for future research on this topic. Given that most health systems, particularly in LMICs like India, are designed to address single conditions, the findings of this study will offer critical insights for transitioning towards a more integrated approach to managing multimorbidity across all age groups.

### Limitations

This study has few limitations. First, as we are collecting data from an already established cohort for cardiometabolic diseases, we do not have data on the additional 11 conditions used to define multimorbidity. While we will inquire about the date of diagnosis for these conditions, this information is subject to recall bias. Considering the methodological limitations of this data, the new data collected during this proposed study will provide an appropriate platform to develop a future multimorbidity cohort in India.

Furthermore, as the study primarily represents an urban population, this cohort is not nationally representative, but it fills a critical gap as no incidence data on multimorbidity is currently available from India. Due to the fellowship’s resource limitations, a nationally representative cohort was not feasible. However, comparing our baseline participants’ characteristics with the LASI 2017–18 data (obesity: 27%, tobacco and alcohol use: ~17%)^35^ shows our findings are within range for urban populations. Nationally representative comparisons are limited due to age differences (LASI: 18+, our study: 40+).

Another limitation is the sample size, which may not be sufficient to do a cluster analysis, as cluster analysis is usually done for a large dataset. However, considering the limited data on multimorbidity from India, this could be a good starting point for such analysis and could possibly inform future studies.

## Supplementary material

10.1136/bmjopen-2025-100853 online supplemental file 1
