# Supplementary material for: Multimorbidity among adult Indians –prevaLence, incidence, risk fActors and economic burdeN: the MILAN cohort study protocol
Source: BMJ Open. 2025 Oct 29;15(10):e100853. doi: 10.1136/bmjopen-2025-100853 (PMC12574355; doi:10.1136/bmjopen-2025-100853)
Supplement: online supplemental file 1 [file bmjopen-15-10-s001.docx]

Supplementary materials

Validated study tools for new data collection

A) Multimorbidity questionnaire

| Disease | Question | Yes/no |
| --- | --- | --- |
| Arthritis | Have you ever been diagnosed with arthritis by a doctor? |  |
|  | In last 12 months have you experienced pain, aching, stiffness or swelling in or around the joints (like arms, hands, legs or feet), which were not related to injury and lasted for more than a month |  |
| Diabetes | Have you ever been diagnosed with high blood sugar (diabetes) by a doctor? |  |
| Hypertension | Have you ever been diagnosed with high blood pressure (hypertension) by a doctor? |  |
| Chronic obstructive Lung disease (COPD) | Have you ever been diagnosed with COPD ( asthma, bronchitis, emphysema) |  |
| Acid peptic disease (gastritis) | In last 12 months, have you been diagnosed with gastritis by a doctor? |  |
| Chronic back ache | In last 12 months, have you been diagnosed with chronic back pain by a doctor? |  |
|  | In last 12 months, have you had continuous back pain for more than 3 weeks? |  |
| Heart disease | Have you ever been diagnosed with angina/heart attack and/or heart disease by a doctor? |  |
|  | Do you experience any pain or discomfort in your chest when you walk uphill or hurry or during normal walking? |  |
| Stroke | Have you ever been diagnosed with stroke by a doctor? |  |
|  | Have you suffered from sudden onset of weakness of your arms, leg or one side of the body for more than 24 hours? |  |
| Vision problem | Do you have difficulty in vision even after wearing glasses? |  |
| Deafness | Do you have difficulty in hearing? |  |
| Dementia | Ask this question to a family member:  Have he/she ever been diagnosed of having dementia by a doctor? |  |
|  | Do he/she have memory problems which hinders activities of daily living? |  |
| Alcohol disorder | Have you visited any doctor because of alcohol disorder/s?  Are you habituated to alcohol? |  |
| Cancer | Have you ever been diagnosed with cancer by a doctor? |  |
| Chronic kidney disease | Have you ever been diagnosed with a long term kidney problem by a doctor? |  |
|  | Have you ever been on dialysis? |  |
| Epilepsy | Have you ever suffered with a sudden onset of seizure/s while at work or rest? |  |
|  | Have you ever been diagnosed with epilepsy by a doctor? |  |
| Thyroid disease | Have you ever been diagnosed with thyroid disease by a doctor? |  |
| Tuberculosis | Do you have tuberculosis?  Are you taking any treatment for TB? |  |
| Other(s) | Do you have any other disease for which you are taking treatment for more than one month?  Specify if yes: |  |

If the answer for any of above disease is yes, ask following questions:

1. When was this diagnosed for the first time? (ask for the year of diagnosis)
2. Have you ever been prescribed any medication for this condition by a doctor?
3. Are you still continuing to take those medicines?
4. If yes, Please name/show the medicines.

As we will have electronic data collection, field workers will be asked to take photographs of medicines and reports, which can be used to ascertain the diagnosis.

B) WHO Disability Assessment Schedule 2.0 (WHODAS 2.0)

Scoring: 0 = No Difficulty, 1 = Mild Difficulty, 2 = Moderate Difficulty, 3 = Severe Difficulty 4 = Extreme Difficulty or Cannot Do


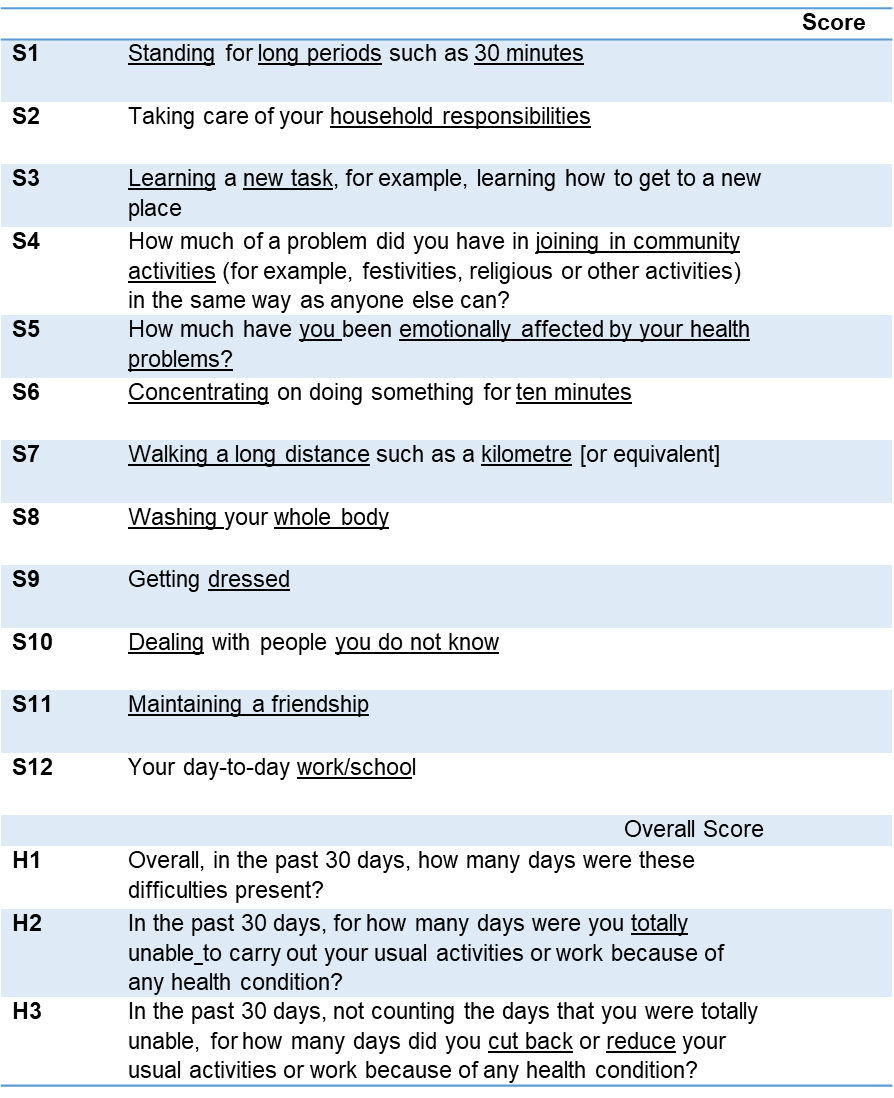


Fried’s Frailty Phenotype scale

| **Criterion** | **Frailty Status** |
| --- | --- |
| **Shrinking** | **Frailty cut point:**  **Baseline:** Self reported unintentional weight loss ≥10lb in previous year  **Follow-up:** Unintentional weight loss ≥5% of previous year’s body weight  *OR*  BMI <18.5kg/m2 |
| **Physical endurance/energy** | *Geriatric Depression Scale:*  *1. Do you feel full of energy?*  *2. During the last 4 weeks how often you rested in bed during day?*  Response options: Every day, every week, once, not at all.  **Frailty cut point:**  No to 1 and every day or every week to 2. |
| **Low physical activity** | *Frequency of mildly energetic, moderately energetic and very energetic physical activity.*  Response options: ≥3 times per week, 1-2 times per week, 1-3 times per month, hardly ever/never  **Frailty cut point:**  Hardly ever/never for very energetic physical activity AND for moderately energetic physical activity. |
| **Weakness** | Hand grip strength in Kg: GRIP-D hand held dynamometer, dominant hand, average of 3 measures.  **Frailty cut point:**  **Grip strength:** lowest 20% (by gender, body mass index)  *Men*  BMI ≤24 ≤29  BMI 24.1–26 ≤30  BMI 26.1–28 ≤30  BMI >28 ≤32  *Women*  BMI ≤23 ≤17  BMI 23.1–26 ≤17.3  BMI 26.1–29 ≤18  BMI >29 ≤21 |
| **Slow walking speed** | Walking time in seconds (usual pace) over 15 feet  **Frailty cut point:**  Slowest 20%, stratified by gender and median standing height.  *Men*  Height ≤173 cm ≥7 seconds  Height >173 cm ≥6 seconds  *Women*  Height ≤159 cm ≥7 seconds  Height >159 cm ≥6 seconds  *OR*  Time to complete “timed up and go test” (TUG)  **Frailty cut point:**  TUG time ≥19 seconds |

**Frail:** ≥3 criteria present; **Intermediate or Pre-Frail:**1 or 2 criteria present; **Robust :** 0 criteria present
