# Supplementary material for: Multimorbidity among adult Indians –prevaLence, incidence, risk fActors and economic burdeN: the MILAN cohort study protocol
Source: BMJ Open. 2025 Oct 29;15(10):e100853. doi: 10.1136/bmjopen-2025-100853 (PMC12574355; doi:10.1136/bmjopen-2025-100853)
Supplement: Supplementary file 2 [file bmjopen-2025-100853.reviewer_comments.pdf]

PEER REVIEW HISTORY

BMJ Open publishes all reviews undertaken for accepted manuscripts. Reviewers are asked to complete a checklist review form and are provided with free text boxes to elaborate on their assessment. These free text comments are reproduced below.

ARTICLE DETAILS

Title (Provisional)

Multimorbidity among Adult Indians - PrevaLence, Incidence, risk fActors and Economic BurdeN: the MILAN cohort study protocol.

Authors

Gupta, Priti; Mohan, Sailesh; McCowan, Colin; Pati, Sanghamitra; Kondal, Dimple; Mohan, Viswanathan; Prabhakaran, Dorairaj

---

VERSION 1 - REVIEW

**Reviewer**	**1**
**Name**	**Dodhia, Hiten**
**Affiliation**	**London Borough of Lambeth, Public Health**
**Date**	**10-May-2025**
**COI**	**None**

Title (page 2 of 26)

Multimorbidity among Adult Indians - PrevaLence, Incidence, risk fActors and Economic BurdeN: the MILAN study protocol.

Abstract (page 3 of 26)

Appears balanced and complete

Background (page 5 of 26)

Rational for the study is given and appears appropriate and comprehensive. This study once completed will fill evidence gaps in the understanding of multiple long-term conditions in the Indian subcontinent. Overall, it appears to be a well-designed and comprehensive look at the incidence of multimorbidity, the longitudinal trends, the common clusters of conditions and the health and economic impact of multimorbidity. This is a protocol paper for a cohort study whose overall aim is to describe multimorbidity and its determinants in a large representative Indian population aged ≥40 years.

Methods (page 5 of 26)

Study population and sampling strategy (page 5 of 26 line 56)

The study population will be drawn from a pre-existing community-based cohort study from two independently sampled cohorts of adults aged 20-99. These cohorts are from two urban centres: Delhi (in north India) and Chennai (in south India) known as the Centre for cArdiometabolic Risk Reduction in South-Asia (CARRS) study 1 & 2.

Study measures (page 6 of 26 line 14)

The authors plan to use both measures already developed in the CARRS study and add some additional measures. These are appropriately described in detail in this section and in table 2. My queries regarding the measures in table 2 are as follows:

Healthcare costs – will only outpatient "expenses" be measured (what about hospital stay, primary or community care expenses)? How will the authors differentiate private and public funded service expenses.

Depression – has the PHQ9 for depression ascertainment been validated in the Indian population?

Quality of life – has the EQ-5D been validated in the Indian population as an appropriate tool for quality-of-life measurement?

The study questionnaires will be translated into Hindi and Tamil – are these the only two languages; is the questionnaire self-administered or will a researcher ask the questions? How will the authors deal with those who cannot read or write?

Study design, participant selection, and recruitment (page 7 of 26 line 30)

Design – this study is using a sample population aged >40 from an existing cohort study (CARRS). Appears to be an appropriate design and selection of cohort. However, the cohort is selected from two urban areas one in North India (Delhi) and one in South India (Chennai). It is unclear from the protocol how truly representative this cohort sample will be of the whole population of India (which is very diverse in terms of ethnicity, language, religions etc.). Figure 1 is mentioned here but not shown in the body of the text – if this is shown on page 20 of 26 (in the appendix) then it is not labelled as figure 1.

Sample size justification (Page 8 0f 26 line 3)

Seems appropriate but statistician needs to review sample size calculations and other statistical methods. I assume the calculations have factored in losses to follow up.

Data collection, management & quality assurance (page 3 of 26 line 36)

This appears to be appropriate.

Biomarker assessments (page 3 of 26 line 55)

Collection, storage, and analysis of biological samples

This appears to be appropriate.

Patient and Public Involvement (page 10 of 26 line 40)

No PPI – this is a bit surprising given the importance of the study. At the very least proper informed consent should be obtained as to the purpose of the study and any findings disseminated to the participating cohort in a language that they can understand.

Baseline demographic details of participants (page 10 of 26 line 44)

Baseline data are presented from the CARRS cohort from which this sample will be drawn. It would be interesting to understand how representative this is of the general population in India – for e.g. the baseline data shows ~58% of people are obese and 13% overweight with only about 15% in the normal weight range – how does this compare with the general population. This could apply to the other characteristics being looked at e.g. tobacco use, alcohol, wealth index etc. and relates to my query above about the representativeness of this cohort to the wider general population.

Statistical analysis plan (page 11 of 26 line 56)

Prevalence: The approach to capturing the current and future impact of disease incidence on physical functioning appears interesting. However, how valid is the use of the SF36 to the Indian population cohort for doing this – has the validity and reliability of the SF36 been tested in the Indian sub-continent including the weighting?

Risk factors: this approach seems appropriate, but a statistician needs to review the analysis proposed.

Incidence: this approach seems appropriate, but a statistician needs to review the analysis proposed.

Health impacts of mLTCs: this approach seems appropriate, but a statistician needs to review the analysis proposed.

Economic impacts of mLTCS: this approach seems appropriate, but a health economist/statistician needs to review the analysis proposed. No health economist appears to be involved in the study design.

DALYs and cluster analysis: this approach seems appropriate, but a statistician needs to review the analysis proposed.

Ethics (page 13 of 26 line 37)

Approval has been obtained but not much details given. For e.g. what is the process for consenting participants in the study? Were participants incentivised for their participation with vouchers or money?

Discussion (page 14 of 26)

The authors highlight the importance of this study to the Indian context. The discussion appropriately covers the rational for doing this study, gaps in evidence that it will fill and

how it may translate into strategies. The discussion also covers the strengths and potential limitations of the study design.

Should the section on the number of long-term conditions that are planned to be included in the study be in the methods section? (lines 24-30 page 14)

The funding for this study should be clearly spelled out not just in abbreviations- “WT/DBT India Alliance”.

References

Appropriate references given.

Appendix/Supplementary data

Page 20 of 26 – figure number and tile missing.

Multimorbidity questionnaire (Page 21 of 26) Is there reference as to how this questionnaire has been validated in the Indian population. How will field workers approach in asking these questions about specific diseases in a non-technical way and ascertain the presence or absence of these diseased – e.g. any visual cues etc.

Page 23 of 26 line 10 – has the WHO disability index been validated in the Indian population?

Page 24 of 26 line 50 has the Fried’s Frailty Phenotype scale been validated in the Indian population?

**Reviewer**	**2**
**Name**	**Lee, Poay Sian Sabrina**
**Affiliation**	**National Healthcare Group Polyclinics, Clinical Research Unit**
**Date**	**02-Jun-2025**
**COI**	**None**

Thank you for the opportunity to review this protocol.

CARRS cohort currently has more than 12,000 participants. I am wondering how many participants aged 40 years and above who fulfil the criteria and are willing to follow up. What happens in longitudinal cohort studies is that participants dropped out or loss to follow up. Are the participants reimbursed for their time during the study visits to complete questionnaires and blood samples?

I noticed that there is substantial number of participants who are illiterate. How do the study team take informed consent? What are the methods of administration of questionnaires? This would affect the rigour and data quality of the study.

I see that blood samples are collected for various laboratory tests. Are the study team planning to store the serum/plasma samples for biobanking purposes?

For disability, is the study team exploring other procedures besides WHODAS 2.0? Perhaps something to do with physical measurements? I guess the same applies to frailty. Fried's frailty phenotype is robust for research setting but how about gait speed or sit to stand test or SPPB?

VERSION 1 - AUTHOR RESPONSE

Reviewer: 1
Dr. Hiten Dodhia, London Borough of Lambeth
Comments to the Author:
Title (page 2 of 26)
Multimorbidity among Adult Indians - PrevaLence, Incidence, risk fActors and Economic BurdeN: the MILAN study protocol.

Abstract (page 3 of 26)
Appears balanced and complete

Response: Thank you for your positive feedback.

Background (page 5 of 26)
Rational for the study is given and appears appropriate and comprehensive. This study once completed will fill evidence gaps in the understanding of multiple long-term conditions in the Indian subcontinent. Overall, it appears to be a well-designed and comprehensive look at the incidence of multimorbidity, the longitudinal trends, the common clusters of conditions and the health and economic impact of multimorbidity. This is a protocol paper for a cohort study whose overall aim is to describe multimorbidity and its determinants in a large representative Indian population aged ≥40 years.
Response: Thank you. We appreciate your acknowledgment of the study's potential contribution to understanding MLTCs in India.

Methods (page 5 of 26)
Study population and sampling strategy (page 5 of 26 line 56)
The study population will be drawn from a pre-existing community-based cohort study from two independently sampled cohorts of adults aged 20-99. These cohorts are from two urban centres: Delhi (in north India) and Chennai (in south India) known as the Centre for cArdiometabolic Risk Reduction in South-Asia (CARRS) study 1 & 2.
Response: Thank you for the observation.

Study measures (page 6 of 26 line 14)
The authors plan to use both measures already developed in the CARRS study and add some additional measures. These are appropriately described in detail in this section

and in table 2. My queries regarding the measures in table 2 are as follows:
Healthcare costs – will only outpatient "expenses" be measured (what about hospital stay, primary or community care expenses)? How will the authors differentiate private and public funded service expenses.

Response: We will focus on out-of-pocket expenditures for both outpatient and inpatient services, along with medicine costs. We also ask about insurance coverage and the nature of the insurance (government vs. private).

Depression – has the PHQ9 for depression ascertainment been validated in the Indian population?
Response: Yes, PHQ-9 has been validated and used extensively in Indian populations, including in our cohort since 2010.
Reference: https://pmc.ncbi.nlm.nih.gov/articles/PMC8155718/

Quality of life – has the EQ-5D been validated in the Indian population as an appropriate tool for quality-of-life measurement?
Response: Yes, EQ-5D has been validated in India and used in our cohort since baseline.
Reference: https://pmc.ncbi.nlm.nih.gov/articles/PMC9936451/

The study questionnaires will be translated into Hindi and Tamil – are these the only two languages; is the questionnaire self-administered or will a researcher ask the questions? How will the authors deal with those who cannot read or write?
Response: The questionnaires are translated into Hindi and Tamil and will be administered by trained field researchers. None of the questionnaires are self-administered, which allows us to effectively include participants who are illiterate.

Study design, participant selection, and recruitment (page 7 of 26 line 30)
Design – this study is using a sample population aged >40 from an existing cohort study (CARRS). Appears to be an appropriate design and selection of cohort. However, the cohort is selected from two urban areas one in North India (Delhi) and one in South India (Chennai). It is unclear from the protocol how truly representative this cohort sample will be of the whole population of India (which is very diverse in terms of ethnicity, language, religions etc.). Figure 1 is mentioned here but not shown in the body of the text – if this is shown on page 20 of 26 (in the appendix) then it is not labelled as figure 1.
Response: We agree that our urban cohort is not nationally representative, but it fills a critical gap as no incidence data on multimorbidity is currently available from India. Due to the fellowship's resource limitations, a nationally representative cohort was not feasible. Figure 1 has now been appropriately labelled and inserted in the main text.

Sample size justification (Page 8 0f 26 line 3)
Seems appropriate but statistician needs to review sample size calculations and other statistical methods. I assume the calculations have factored in losses to follow up.

Response: Yes, we have factored in a 25% loss to follow-up in our sample size calculations, which is detailed in Table 2

Data collection, management & quality assurance (page 3 of 26 line 36)
This appears to be appropriate.

Response: Thank you

Biomarker assessments (page 3 of 26 line 55)
Collection, storage, and analysis of biological samples
This appears to be appropriate.

Response: Thank you

Patient and Public Involvement (page 10 of 26 line 40)
No PPI – this is a bit surprising given the importance of the study. At the very least proper informed consent should be obtained as to the purpose of the study and any findings disseminated to the participating cohort in a language that they can understand.
Response: We obtain proper written informed consent and have now added a clarification regarding this process in the manuscript. We will also ensure dissemination of findings to participants in their local language.

Baseline demographic details of participants (page 10 of 26 line 44)
Baseline data are presented from the CARRS cohort from which this sample will be drawn. It would be interesting to understand how representative this is of the general population in India – for e.g. the baseline data shows ~58% of people are obese and 13% overweight with only about 15% in the normal weight range – how does this compare with the general population. This could apply to the other characteristics being looked at e.g. tobacco use, alcohol, wealth index etc. and relates to my query above about the representativeness of this cohort to the wider general population.
Response: We acknowledge that our cohort from metropolitan cities is not nationally representative. However, comparing with the LASI 2017–18 data (obesity: 27%, tobacco and alcohol use: ~17%) shows our findings are within range for urban populations. Nationally representative comparisons are limited due to age differences (LASI: 18+, our study: 40+)

Statistical analysis plan (page 11 of 26 line 56)
Prevalence: The approach to capturing the current and future impact of disease incidence on physical functioning appears interesting. However, how valid is the use of the SF36 to the Indian population cohort for doing this – has the validity and reliability of the SF36 been tested in the Indian sub-continent including the weighting?
Risk factors: this approach seems appropriate, but a statistician needs to review the analysis proposed.
Incidence: this approach seems appropriate, but a statistician needs to review the analysis proposed.

Health impacts of mLTCs: this approach seems appropriate, but a statistician needs to review the analysis proposed.
Economic impacts of mLTCS: this approach seems appropriate, but a health economist/statistician needs to review the analysis proposed. No health economist appears to be involved in the study design.
DALYs and cluster analysis: this approach seems appropriate, but a statistician needs to review the analysis proposed.

Response: We are not directly applying the SF-36 in our cohort; instead, we are using the disease weights that were developed in the U.S. based on SF-36 data.
We confirm that statisticians and a health economist have been involved in reviewing the analysis plan.

Ethics (page 13 of 26 line 37)
Approval has been obtained but not much details given. For e.g. what is the process for consenting participants in the study? Were participants incentivised for their participation with vouchers or money?
Response: Participants were not given any incentives. This is a long-standing cohort and participation is based on trust and engagement built over time.

Discussion (page 14 of 26)
The authors highlight the importance of this study to the Indian context. The discussion appropriately covers the rational for doing this study, gaps in evidence that it will fill and how it may translate into strategies. The discussion also covers the strengths and potential limitations of the study design.
Should the section on the number of long-term conditions that are planned to be included in the study be in the methods section? (lines 24-30 page 14)
The funding for this study should be clearly spelled out not just in abbreviations- “WT/DBT India Alliance”.
Response: We have mentioned the long term conditions in the supplementary file as part of multimorbidity questionnaire. The funding source has now been fully spelled out as “Wellcome Trust/Department of Biotechnology (WT/DBT) India Alliance”.

References
Appropriate references given.

Response: Thank you

Appendix/Supplementary data
Page 20 of 26 – figure number and tile missing.
Response: Figure 1 has been added and appropriately labelled.

Multimorbidity questionnaire (Page 21 of 26) Is there reference as to how this questionnaire has been validated in the Indian population. How will field workers approach in asking these questions about specific diseases in a non-technical way and ascertain the presence or absence of these diseased – e.g. any visual cues etc.

Response: Yes this questionnaire is validated in Indian population. Trained field staff will use simple, non-technical language and visual cues where appropriate.
Reference: Pati S, Hussain MA, Swain S, Salisbury C, Metsemakers JFM, Knottnerus JA, et al. Development and Validation of a Questionnaire to Assess Multimorbidity in Primary Care: An Indian Experience. Biomed Res Int. 2016;2016:1–9.

Page 23 of 26 line 10 – has the WHO disability index been validated in the Indian population?
Response: Yes.
Reference:https://pmc.ncbi.nlm.nih.gov/articles/PMC7905154/#:~:text=The%20strengths%20of%20the%20current,in%20a%20multiethnic%20Asian%20population.

Page 24 of 26 line 50 has the Fried's Frailty Phenotype scale been validated in the Indian population?
Response: Yes. It has been extensively used and validated in Indian settings.
Reference: https://pmc.ncbi.nlm.nih.gov/articles/PMC11668389/

Reviewer: 2
Dr. Poay Sian Sabrina Lee, National Healthcare Group Polyclinics
Comments to the Author:
Thank you for the opportunity to review this protocol.
CARRS cohort currently has more than 12,000 participants. I am wondering how many participants aged 40 years and above who fulfil the criteria and are willing to follow up. What happens in longitudinal cohort studies is that participants dropped out or loss to follow up. Are the participants reimbursed for their time during the study visits to complete questionnaires and blood samples?
Response: Thank you for this important observation. Among the CARRS cohort, a substantial proportion (approximately 9,000 participants) are aged ≥40 years. Based on prior follow-up rounds, we maintain a follow-up rate of approximately 70%, with around 30% attrition primarily due to mortality.

Participants are not reimbursed financially for their time. However, the long-standing relationship and trust built over the past decade with our cohort participants have played a vital role in maintaining high response rates and continued engagement.

I noticed that there is substantial number of participants who are illiterate. How do the study team take informed consent? What are the methods of administration of questionnaires? This would affect the rigour and data quality of the study.
Response: We fully recognize the importance of ensuring ethical conduct and data quality, particularly for participants with limited literacy. Our process includes the following steps:

- *Informed consent is obtained through a standardized procedure conducted by trained field researchers. They explain the consent form verbally in the participant's local language, detailing the purpose, procedures, potential risks and benefits, and participant rights (including withdrawal at any time).*

- *If preferred by the participant, a trusted family member may assist in interpreting the information.*

- *Consent is documented through a thumb impression and witnessed by an impartial third party.*

All questionnaires are administered face-to-face by trained enumerators who read each question aloud and accurately record the participant's responses. This method ensures that participants fully understand the questions and minimizes response bias due to literacy barriers. To ensure data quality and consistency, our team implements regular training sessions, field supervision, and spot-checks.

I see that blood samples are collected for various laboratory tests. Are the study team planning to store the serum/plasma samples for biobanking purposes?
Response: Yes, as part of this well-established cohort, we have developed infrastructure to store laboratory aliquots, including serum and plasma, for future research purposes. This biobanking effort enables longitudinal biological investigations over time.

For disability, is the study team exploring other procedures besides WHODAS 2.0? Perhaps something to do with physical measurements? I guess the same applies to frailty. Fried's frailty phenotype is robust for research setting but how about gait speed or sit to stand test or SPPB?
Response: Yes, in addition to WHODAS 2.0 for disability assessment, we are incorporating physical performance measures including:

Gait speed

Grip strength test

These are part of our frailty assessment protocol to enhance the objectivity and robustness of physical function data. While Fried's frailty phenotype is our primary measure, the inclusion of these physical assessments allows for broader functional characterization in our population.

VERSION 2 - AUTHOR RESPONSE

Reviewer: 1
Dr. Hiten Dodhia, London Borough of Lambeth
Comments to the Author:
Title (page 2 of 26)
Multimorbidity among Adult Indians - PrevaLence, Incidence, risk fActors and Economic BurdeN: the MILAN study protocol.

Study measures (page 6 of 26 line 14)
The authors plan to use both measures already developed in the CARRS study and add some additional measures. These are appropriately described in detail in this section

and in table 2. My queries regarding the measures in table 2 are as follows: Healthcare costs – will only outpatient "expenses" be measured (what about hospital stay, primary or community care expenses)? How will the authors differentiate private and public funded service expenses.

Response: We will focus on out-of-pocket expenditures for both outpatient and inpatient services, along with medicine costs. We also ask about insurance coverage and the nature of the insurance (government vs. private). We have added this information in line 202-204.

Depression – has the PHQ9 for depression ascertainment been validated in the Indian population?
Response: Yes, PHQ-9 has been validated and used extensively in Indian populations, including in our cohort since 2010. We have added this information in line number 181-183.

Quality of life – has the EQ-5D been validated in the Indian population as an appropriate tool for quality-of-life measurement?
Response: Yes, EQ-5D has been validated in India and used in our cohort since baseline. We have added this information in line number 181-183.

The study questionnaires will be translated into Hindi and Tamil – are these the only two languages; is the questionnaire self-administered or will a researcher ask the questions? How will the authors deal with those who cannot read or write?
Response: The questionnaires are translated into Hindi and Tamil and will be administered by trained field researchers. None of the questionnaires are self-administered, which allows us to effectively include participants who are illiterate. We have added this information in line number 243-245.

Study design, participant selection, and recruitment (page 7 of 26 line 30)
Design – this study is using a sample population aged >40 from an existing cohort study (CARRS). Appears to be an appropriate design and selection of cohort. However, the cohort is selected from two urban areas one in North India (Delhi) and one in South India (Chennai). It is unclear from the protocol how truly representative this cohort sample will be of the whole population of India (which is very diverse in terms of ethnicity, language, religions etc.). Figure 1 is mentioned here but not shown in the body of the text – if this is shown on page 20 of 26 (in the appendix) then it is not labelled as figure 1.
Response: We agree that our urban cohort is not nationally representative, but it fills a critical gap as no incidence data on multimorbidity is currently available from India. Due to the fellowship's resource limitations, a nationally representative cohort was not feasible. We have added these lines in the limitation section of discussion in line number 458-465. Figure 1 has now been appropriately labelled and inserted in the main text.

Baseline demographic details of participants (page 10 of 26 line 44)
Baseline data are presented from the CARRS cohort from which this sample will be drawn. It would be interesting to understand how representative this is of the general population in India – for e.g. the baseline data shows ~58% of people are obese and 13% overweight with only about 15% in the normal weight range – how does this compare with the general population. This could apply to the other characteristics being looked at e.g. tobacco use, alcohol, wealth index etc. and relates to my query above about the representativeness of this cohort to the wider general population.
Response: We acknowledge that our cohort from metropolitan cities is not nationally representative. However, comparing with the LASI 2017–18 data (obesity: 27%, tobacco and alcohol use: ~17%) shows our findings are within range for urban populations. Nationally representative comparisons are limited due to age differences (LASI: 18+, our study: 40+). We have added these lines in the limitation section of discussion in line number 458-465

Statistical analysis plan (page 11 of 26 line 56)
Prevalence: The approach to capturing the current and future impact of disease incidence on physical functioning appears interesting. However, how valid is the use of the SF36 to the Indian population cohort for doing this – has the validity and reliability of the SF36 been tested in the Indian sub-continent including the weighting?
Risk factors: this approach seems appropriate, but a statistician needs to review the analysis proposed.
Incidence: this approach seems appropriate, but a statistician needs to review the analysis proposed.
Health impacts of mLTCs: this approach seems appropriate, but a statistician needs to review the analysis proposed.
Economic impacts of mLTCS: this approach seems appropriate, but a health economist/statistician needs to review the analysis proposed. No health economist appears to be involved in the study design.
DALYs and cluster analysis: this approach seems appropriate, but a statistician needs to review the analysis proposed.

Response: We are not directly applying the SF-36 in our cohort; instead, we are using the disease weights that were developed in the U.S. based on SF-36 data. We have added this information in line 322-324.
We confirm that statisticians and a health economist have been involved in reviewing the analysis plan.

Ethics (page 13 of 26 line 37)
Approval has been obtained but not much details given. For e.g. what is the process for consenting participants in the study? Were participants incentivised for their participation with vouchers or money?
Response: Participants were not given any incentives. This is a long-standing cohort and participation is based on trust and engagement built over time. We have added this information is line 276-277

Discussion (page 14 of 26)
The authors highlight the importance of this study to the Indian context. The discussion appropriately covers the rational for doing this study, gaps in evidence that it will fill and how it may translate into strategies. The discussion also covers the strengths and potential limitations of the study design.
Should the section on the number of long-term conditions that are planned to be included in the study be in the methods section? (lines 24-30 page 14)
The funding for this study should be clearly spelled out not just in abbreviations- "WT/DBT India Alliance".
Response: We have added the conditions in line 187-193. We have added the number of long term conditions in the method section. The funding source has now been fully spelled out as "Wellcome Trust/Department of Biotechnology (WT/DBT) India Alliance".

Multimorbidity questionnaire (Page 21 of 26) Is there reference as to how this questionnaire has been validated in the Indian population. How will field workers approach in asking these questions about specific diseases in a non-technical way and ascertain the presence or absence of these diseased – e.g. any visual cues etc.
Response: Yes this questionnaire is validated in Indian population. Trained field staff will use simple, non-technical language and visual cues where appropriate. We have added this information in line 185-187

Page 23 of 26 line 10 – has the WHO disability index been validated in the Indian population?
Response: Yes. We have added this information in line 183-185.

Page 24 of 26 line 50 has the Fried's Frailty Phenotype scale been validated in the Indian population?
Response: Yes. We have added this information in line 183-185

Reviewer: 2
Dr. Poay Sian Sabrina Lee, National Healthcare Group Polyclinics
Comments to the Author:
Thank you for the opportunity to review this protocol.
CARRS cohort currently has more than 12,000 participants. I am wondering how many participants aged 40 years and above who fulfil the criteria and are willing to follow up. What happens in longitudinal cohort studies is that participants dropped out or loss to follow up. Are the participants reimbursed for their time during the study visits to complete questionnaires and blood samples?
Response: Thank you for this important observation. Among the CARRS cohort, a substantial proportion (approximately 9,000 participants) are aged ≥40 years. Based on prior follow-up rounds, we maintain a follow-up rate of approximately 70%, with around 30% attrition primarily due to mortality. We have added this information in line 212-215.

Participants are not reimbursed financially for their time. However, the long-standing relationship and trust built over the past decade with our cohort participants have played a vital role in maintaining high response rates and continued engagement. We have added this information in line 217-219.

I noticed that there is substantial number of participants who are illiterate. How do the study team take informed consent? What are the methods of administration of questionnaires? This would affect the rigour and data quality of the study.
Response: We fully recognize the importance of ensuring ethical conduct and data quality, particularly for participants with limited literacy. Our process includes the following steps:

- *Informed consent is obtained through a standardized procedure conducted by trained field researchers. They explain the consent form verbally in the participant's local language, detailing the purpose, procedures, potential risks and benefits, and participant rights (including withdrawal at any time).*

- *If preferred by the participant, a trusted family member may assist in interpreting the information.*

- *Consent is documented through a thumb impression and witnessed by an impartial third party.*

All questionnaires are administered face-to-face by trained enumerators who read each question aloud and accurately record the participant's responses. This method ensures that participants fully understand the questions and minimizes response bias due to literacy barriers. To ensure data quality and consistency, our team implements regular training sessions, field supervision, and spot-checks. We have added this information in line 260-276.

I see that blood samples are collected for various laboratory tests. Are the study team planning to store the serum/plasma samples for biobanking purposes?
Response: Yes, as part of this well-established cohort, we have developed infrastructure to store laboratory aliquots, including serum and plasma, for future research purposes. This biobanking effort enables longitudinal biological investigations over time. We have added this information in line 303-305.

For disability, is the study team exploring other procedures besides WHODAS 2.0? Perhaps something to do with physical measurements? I guess the same applies to frailty. Fried's frailty phenotype is robust for research setting but how about gait speed or sit to stand test or SPPB?
Response: Yes, in addition to WHODAS 2.0 for disability assessment, we are incorporating physical performance measures including: Gait speed and Grip strength test
These are part of our frailty assessment protocol to enhance the objectivity and robustness of physical function data. While Fried's frailty phenotype is our primary

measure, the inclusion of these physical assessments allows for broader functional characterization in our population. We have added this information in line 197-202.
